# Prevalence of pressure injury on the medical wards of public general hospitals in Kuwait: a national cross-sectional study

**DOI:** 10.1186/s12913-024-10615-x

**Published:** 2024-02-07

**Authors:** Talal ALFadhalah, Marjan Lari, Gheed Al Salem, Shaimaa Ali, Hamad Al Kharji, Hossam Elamir

**Affiliations:** 1 Quality and Accreditation Directorate, Ministry of Health, Kuwait City, Kuwait; 2 Nursing Department, Quality and Accreditation Directorate, Ministry of Health, Kuwait City, Kuwait; 3 Accreditation Affairs Department, Quality and Accreditation Directorate, Ministry of Health, Kuwait City, Kuwait; 4 Research and Technical Support Department, Quality and Accreditation Directorate, Ministry of Health, Kuwait City, Kuwait

**Keywords:** Bedsores, Braden scale, Decubitus ulcers, Hospital-acquired complications, Patient safety, Risk assessment

## Abstract

**Background:**

Pressure injury is a severe problem that can significantly impact a patient’s health, quality of life, and healthcare expenses. The prevalence of pressure injuries is a widely used clinical indicator of patient safety and quality of care. This study aims to address the research gap that exists on this topic in Kuwait by investigating the prevalence of pressure injuries and preventive measures on the medical wards of the country’s public general hospitals.

**Methods:**

A cross-sectional research design was adopted to measure the point prevalence of pressure injuries on 54 medical wards in the public general hospitals. Data, including variables pertaining to hospitals, patients, pressure injuries and preventive practices, were collected using an online form. The data were processed and analysed using Microsoft Excel and SPSS 23 (α level = 0.05). Analysis provided an overview of patient, pressure injury characteristics and preventive measures, and the relationships between the patient and pressure injury characteristics and the prevalence of pressure injuries. A model for predicting the determinants of pressure injury prevalence was constructed from a linear regression analysis.

**Results:**

The mean national prevalence of pressure injury was 17.6% (95% CI: 11.3–23.8). Purely community-acquired pressure injuries represent the majority of pressure injuries nationally (58.1%). Regarding preventive measures, “pressure injury assessment on admission” has been provided to 65.5% of patients. Correlation analysis revealed that the only statistically significant correlation with the prevalence of hospital-acquired pressure injury was “pressure injury assessment on admission”, which was strongly negative (ρ = −0.857). Therefore, this was the only variable included in the regression analysis as a predictor of pressure injury prevalence (Beta = 0.839). The results showed many statistically significant differences between hospitals with respect to the variables studied.

**Conclusions:**

The national pressure injury prevalence is high compared to the global rate. The higher percentage of purely community-acquired pressure injuries requires particular attention. Many risk factors for the development of pressure injuries are public health concerns, and effective mitigating strategies are needed. Further research is required to assess the knowledge, attitude, and behaviour of nurses with respect to pressure injuries, and to evaluate preventive and management practices.

**Supplementary Information:**

The online version contains supplementary material available at 10.1186/s12913-024-10615-x.

## Background

Pressure injuries (PIs) are one of the most commonly encountered types of chronic wounds [1, 2]. A PI is the localised damage caused by persistent or severe pressure with contributions from shear and friction forces, which usually occurs to the skin and underlying soft tissue over a bony prominence or under medical or other devices [3, 4]. Hospital-acquired PIs (HAPIs) are globally considered “never events” [5, 6], for being largely preventable and reducible in their severity by using a multifaceted approach [7, 8]. Of all health conditions, PI currently ranks among the highest in terms of cost, mortality and morbidity rates and prolonged hospitalisation [3, 6, 9, 10].

Data show that PIs can develop within a period of 1 to 6 h [11]. The prompt and accurate identification of at-risk individuals is therefore paramount so that preventive measures can be implemented [6, 12]. The Braden Risk Assessment Tool [13] is one of the suggested validated tools for assessing PI risk among adult populations [6]. Risk factors for developing PIs include advanced age, spinal cord injury, decreased sensory perception, unfavourable skin microclimate, faecal and urinary incontinence, poor nutritional status, limited activity and impaired mobility and increased friction and shear forces [7, 12, 14, 15].

Accurate staging of PIs is essential for appropriate assessment, management and prevention [4, 10, 15]. The six-category staging system adopted by the National Pressure Ulcer Advisory Panel (NPUAP) in conjunction with the European Pressure Advisory Panel (EPUAP) and the Pan Pacific Pressure Injury Alliance (PPPIA) is widely used [4]. Stage 1 is characterised by mild to non-blanchable erythema, which develops into severe tissue loss and exposure of underlying structures (Stage 4). The presence of slough or eschar can hinder accurate staging (unstageable PI), and deep discolouration indicates damage at deeper tissue levels (deep-tissue PI).

The prevalence of PIs is a widely used clinical indicator for the standard of care, and has been shown to have important implications for basic nursing, patient safety and quality outcomes [6, 10, 16]. A recent systematic review and meta-analysis reported a global total PI prevalence—in hospitals—of 12.8% (95% CI: 11.8–13.9) and a global HAPIs prevalence of 8.4% (95% CI: 7.6–9.3) [17].

Historically, preventing PIs has been a significant nursing challenge [18–20]. Nurses are the principal implementers of PI-prevention strategies and measures [6, 20, 21]. However, many clinicians and managers believe that PI development is a failure of the entire healthcare system rather than suboptimal nursing care [6, 21, 22].

The significance of PI prevalence, nurses’ knowledge, and attitudes towards PI prevention in effective management cannot be overstated. However, a research gap on this topic exists in Kuwait. Thus, the Quality and Accreditation Directorate at the Ministry of Health in Kuwait launched a national research project with the aim of determining the prevalence rate of PIs and prevention strategies implemented in hospitals, and to examine the knowledge and attitude of nurses towards preventing PIs in hospitalised patients. This research is expected to contribute invaluable insights to the field and enhance PI prevention practices in healthcare settings in Kuwait.

Given the size of the research datasets, this article focuses on the prevalence of PIs and preventive measures. Subsequent articles will report on the assessment of nurses’ knowledge and their attitudes towards PI prevention, and explore the relationship of these two aspects with current PI prevention practices and PI prevalence. Here, we evaluate the extent of PIs on the medical wards of the public general hospitals in Kuwait.

The aim of this study was to investigate the prevalence of PIs and preventive measures on the medical wards of the public general hospitals in Kuwait. Our objectives were to identify the characteristics of patients with PIs, the characteristics and prevalence rates of PIs and the characteristics of the implemented PI preventive measures. We also sought to determine whether there were any differences between the studied hospitals regarding PI-diagnosed patients, PI characteristics and prevalence rates, and the implemented PI preventive measures. Finally, we identified the predictors of the prevalence rate of PIs.

## Methods

### Study design and setting

A cross-sectional descriptive research design was adopted to measure PI point prevalence. Seven health regions manage the different care levels of the public health system in Kuwait. Each health region is allocated one public general hospital, which provides secondary care [23]. The public health system in Kuwait is owned, regulated, managed and operated by the Ministry of Health. The study was conducted on the 54 medical wards of the seven public general hospitals in Kuwait. The Strengthening the Reporting of Observational studies in Epidemiology (STROBE) checklist [24] was followed in the reporting of this observational study.

### Research tools

We used the online “Data Collection Form for Prevalence Rate of Pressure Injury” (Supplementary file 1). After reviewing relevant literature [25], we followed Chaudhary and Israel’s [26] approach in the development and testing of the tool. This approach provides a simple methodology for insider and field testing of the data collection tool which results in rapid improvement of its validity and reliability. The data collection tool was created on Google Forms. Google Forms is a free online tool from Google which allows users to create forms, surveys, and quizzes as well as to collaboratively edit and share the forms with other people. Then, the tool was thoroughly reviewed against the Centres for Disease Control and Prevention’s Question Appraisal System (QAS-99) [27] to identify and fix miscommunication and other types of problems with tool contents. The content validity of the tool was confirmed by a panel of three PI and wound management experts. The tool was field-tested by conducting a pilot study on 10% of patient files at the hospitals to ensure the clarity of the tool and to identify obstacles to the data collection process. Every two data collectors crosschecked their collected data to confirm the reliability of the tool. The collected data during the pilot phase were not included in the study. Notably, the tool could be completed in 10 to 15 min for each patient.

The tool (Supplementary file 1) included the following variables: hospital name, ward number, patient’s age, sex, weight, height, date of admission, date of PI onset, mobility condition/activity, location and number of PIs, hospital or community acquisition, stage, number and type of comorbidities. The data collector also was required to indicate in the tool whether the Braden scale assessment was documented and its score, medical device was involved in the PI development and whether preventive measures were provided.

### Sampling and data collection

A total population sampling technique was utilised and this applied to all patients’ files with active current admission to adult medical wards on the day data was collected. We excluded files of patients who were physically located on medical wards but admitted by non-medical speciality (e.g., surgical or orthopaedic). Patients were not interviewed. The study did not include examining patients, intervening in their treatment, giving them medications, or performing investigations other than the agreed-upon management plans which were approved by the treating team. The study did not include any patient-identifiable information.

We selected medical wards because they have the largest number of inpatient beds, and their patients typically have more co-morbidities and stay admitted for a longer duration. After we had explained the value and potential benefits of the study to the hospital and nursing directors, we obtained their permission to facilitate the process. All nurses on medical wards—whose nursing notes were reviewed—consented to participate after we had explained the value and potential benefits of the study to them.

After consultation with their directors, 40 quality department nurses were selected based on their availability and workload. They were assigned to collect data after they had received the required training and been tested for competency.

Data were collected on 13 December 2022 from open patient files. Data collectors were required to search the medical files of all patients with PIs to find documented information about the aforementioned variables. Then they document their findings in the online form (Supplementary file 1).

### Data management and analysis

Data were processed and cleaned using Excel (Microsoft) and analysed using SPSS 23 (α level = 0.05). The Kolmogorov–Smirnov and Shapiro–Wilk tests were used to check the data normality. The analysis of the quantitative data included univariate descriptive analyses (frequencies, percentages, means, standard deviations, confidence intervals, medians, and interquartile ranges). The analysis also included bivariate analyses (Chi-square tests, ANOVA F-tests) to examine how trends in the patient and PI characteristics differ across hospitals and to investigate the relationships between the patient and PI characteristics and prevalence. Non-parametric tests (Kruskal–Wallis test, Spearman’s correlation) were used if violations of assumptions hindered the use of parametric testing.

We conducted a linear regression analysis to construct a model to help predict the determinants of PI prevalence and develop actionable strategies. Independent variables with statistically significant (*p* ≤.05) correlation coefficients ≥ 0.100 in the correlational analysis were included in the regression model.

## Results

Of the 1,186 patients admitted to 54 medical wards at the seven public general hospitals in Kuwait, 203 (17.1%) were found to have PIs. The data were collected for all of the 203 patients with PIs (100%).

### Descriptive statistics of patients with PIs

Table 1 shows the descriptive statistics of patients with PIs. The sample was almost equally distributed between males (49.8%) and females (50.2%). Slightly less than three-quarters of the sample belonged to two age groups: 56–70 (30.5%) and 71–85 (43.3%) years. Only 26 patients (12.8%) had their weight and height documented. Of these, 38.5% were overweight. Almost half of the patients had stayed for between one week and three months in their current admission. Most of the patients (85.2%) had two co-morbidities; chronic disease (100%) and immobility/reduced mobility (98.5%) were the most frequent. Only 37 patients (18.2%) had Braden scale assessment documented in their files. Of these, almost one-quarter (24.3%) were found to have moderate risk, and slightly more than two-thirds were found to have high risk (32.4%) or severe risk (37.8%).

Table 1 also shows the comparison between the seven hospitals for the aforementioned statistics. There are statistically significant differences between hospitals regarding sex (*p* =.027), median length of stay (*p* <.001), number of co-morbidities (*p* <.001), some types of co-morbidities (sepsis and other co-morbidities, *p* <.001), mobility condition/activity (*p* =.050), and Braden scale assessment documentation and score (*p* <.001).

**Table 1:** Descriptive statistics of patients with PIs

	n (%)		*p*
**Patients with PI**	203	(100.0)	
**Sex(***n* = **203,** %**=100)**			0.027^†^
Male	101	(49.8)	
Female	102	(50.2)	
**Age distribution in years(***n* = **203,** %**=100)**			0.245^†^
16–25	5	(2.5)	
26–40	11	(5.4)	
41–55	15	(7.4)	
56–70	62	(30.5)	
71–85	88	(43.3)	
86–100	22	(10.8)	
Median [IQR]	73	[63–80]	0.253^‡^
**BMI distribution(***n* = **26,** %**=12.8)**			0.218^†^
Underweight (< 18.5)	2	(7.7)	
Normal (18.5– <25)	6	(23.1)	
Overweight (25– <30)	10	(38.5)	
Obesity class I (30– <35)	2	(7.7)	
Obesity class II (35– <40)	4	(15.4)	
Obesity class III (≥ 40)	2	(7.7)	
Mean [SD]	29.1	[6.8]	0.402*
**Length of stay distribution in days(***n* = **203,** %**=100)**			0.194^†^
0–7	22	(10.8)	
8–30	48	(23.6)	
31–90	50	(24.6)	
91–180	31	(15.3)	
181–365	30	(14.8)	
> 365	22	(10.8)	
Median [IQR]	54	[21–185]	< 0.001^‡^
**Number of co-morbidities(***n* = **203,** %**=100)**			< 0.001^†^
1	3	(1.5)	
2	173	(85.2)	
3	25	(12.3)	
4	2	(1.0)	
Median [IQR]	2	[2–2]	< 0.001^‡^
**Co-morbidities** ^**1**^			
Chronic disease	203	(100.0)	
Immobility/reduced mobility	200	(98.5)	0.277^†^
Sepsis	15	(7.4)	< 0.001^†^
Spinal cord injury	3	(1.5)	0.791^†^
Dehydration	1	(0.5)	0.307^†^
Vasopressor infusion	1	(0.5)	0.472^†^
Others	9	(4.4)	< 0.001^†^
**Mobility condition/ Activity(***n* = **203,** %**=100)**			0.050^†^
Immobile	195	(96.1)	
Mobile with assistance	8	(3.9)	
**Braden scale assessment score documented(***n* = **203,** %**=100)**			< 0.001^†^
No	166	(81.8)	
Yes	37	(18.2)	
**Braden scale score range(***n* = **37,** %**=18.2)**			< 0.001^†^
Low risk (19–23)	1	(2.7)	
Mild risk (15–18)	1	(2.7)	
Moderate risk (13–14)	9	(24.3)	
High risk (10–12)	12	(32.4)	
Severe risk (≤ 9)	14	(37.8)	
Mean [SD]	10.8	[3.0]	< 0.001*

n: number; %: percentage; *p*: *p*-value to determine differences between the seven hospitals as regard to the studied variables (statistically significant at *p* ≤.05); IQR: inter-quartile range; SD: standard deviation; BMI: body mass index in kg/m^2^ [28]; 1: Multiple answers are allowed; Statistical test used to determine statistical significance: † Chi-square test; ‡ Kruskal–Wallis test; * ANOVA F-test

### Descriptive statistics of PIs

Table 2 shows the descriptive statistics of the PIs. The mean national prevalence of PIs was 17.6% (95% CI: 11.3–23.8); in the seven individual hospitals it ranged from 8.6 to 26.6%. Purely community-acquired PIs (CAPIs) accounted for the majority of PIs in four of the seven hospitals (hospitals 2, 3, 5 and 7) and at the national level (58.1%). In other words, the mean national prevalence of CAPIs was 11.9% (95% CI: 5.6–18.3). This indicates that the mean national prevalence of HAPIs (6.7%) is notably lower than the national rate of all PIs. The differences between hospitals are statistically significant (*p* ≤.001) regarding the prevalence of PIs, the prevalence of HAPIs and the number of CAPIs versus HAPIs.

PI onset date was documented for only 178 patients (87.7%). Of the 63 PIs acquired in hospital, two-fifths (*n* = 26; 41.3%) developed after one month of admission. The differences between hospitals in this regard are statistically significant (*p* <.001). Although the PIs persisted for a median of 49 days (IQR: 16.5–171.8), 7.9% of PIs lasted longer than 1 year. Again, the differences between hospitals regarding PI duration groups (*p* =.003) and the median (*p* <.001) were statistically significant.

Table 2 also shows that most of the PIs (88.7%) were not related to the use of medical devices. This was the case at all hospitals except hospital 4. Of the 200 documentations of PI staging, two-thirds (66.5%) were recorded as stage 1 or 2. Three-quarters (73.4%) of the patients had only one PI. The three most common anatomical sites for PIs were the sacrum (82.8%), buttocks (13.8%) and heels (13.3%). Unlike PI stage categories, the differences between hospitals in regard to the number of PIs per patient and their anatomical sites (except heels) were not statistically significant.

**Table 2:** Descriptive statistics of PIs

	n (%)		*p*
**All admitted patients**	1186	(100.0)	
**PI is(***n* = **203,** %**=100)**			< 0.001^†^
Community-acquired (CA)	118	(58.1)	
Hospital-acquired (HA)	74	(36.5)	
Community & hospital-acquired	11	(5.4)	
**PI prevalence [CI]**	17.6%	[11.3–23.8]	< 0.001^†^
**HAPI prevalence [CI]**	6.7%	[3.2–10.2]	0.001^†^
**CAPI prevalence [CI]**	11.9%	[5.6–18.3]	< 0.001^†^
**Days till PI developed(***n* = **178,** %**=87.7)**			< 0.001^†^
Before/on admission	115	(64.6)	
1–7	14	(7.9)	
8–30	23	(12.9)	
31–90	19	(10.7)	
91–180	3	(1.7)	
> 180	4	(2.2)	
Median [IQR] (for HAPI)	18	[8.0–50.0]	0.553^‡^
**PI duration(***n* = **178,** %**=87.7)**			0.003^†^
0–7	24	(13.5)	
8–30	48	(27.0)	
31–90	43	(24.2)	
91–180	26	(14.6)	
181–365	21	(11.8)	
> 365	16	(7.9)	
Median [IQR]	49	[16.5–171.8]	< 0.001^‡^
**PI is related to medical device(***n* = **203,** %**=100)**			< 0.001^†^
No	180	(88.7)	
Yes	23	(11.3)	
**Worst-stage PI distribution(***n* = **200,** %**=98.5)**			< 0.001^†^
Stage 1	45	(22.5)	
Stage 2	88	(44.0)	
Stage 3	45	(22.5)	
Stage 4	19	(9.5)	
Suspected deep tissue injury	3	(1.5)	
**Number of PI anatomic locations(***n* = **203,** %**=100)**			0.491^†^
1	149	(73.4)	
2	36	(17.7)	
3	15	(7.4)	
4	2	(1.0)	
7	1	(0.5)	
Median [IQR]	1	[1.0–2.0]	0.244^‡^
**PI anatomic site distribution** ^**1**^			
Sacrum	168	(82.8)	0.287^†^
Buttocks	28	(13.8)	0.253^†^
Heels	27	(13.3)	0.026^†^
Back	24	(11.8)	0.640^†^
Thigh	7	(3.4)	0.799^†^
Lower limbs	7	(3.4)	0.883^†^
Hips	6	(3.0)	0.397^†^
Elbow	5	(2.5)	0.796^†^
Back of head	3	(1.5)	0.358^†^
Anal region	1	(0.5)	0.902^†^
Other sites	5	(2.5)	0.194^†^

n: number; %: percentage; *p*: *p*-value to determine differences between the seven hospitals as regard to the studied variables (statistically significant at *p* ≤.05); HAPI: hospital-acquired pressure injury; CAPI: community-acquired pressure injury; CI: 95% confidence interval; IQR: inter-quartile range; 1: Multiple answers are allowed; Statistical test used to determine statistical significance: † Chi-square test; ‡ Kruskal–Wallis test

The national prevalence of PIs, HAPIs and CAPIs are the means of the seven hospitals’ prevalence values.

### Preventive measures provided

In this study, patients with PIs receiving three preventive measures formed the largest group (24.1%), followed by patients that received four (21.7%; Table 3). The median value of the number of PI preventive measures provided to patients with PIs was three (IQR = 2–4). The differences between hospitals in the number of preventive measures provided per patient (*p* <.001) and its median (*p* =.019) were statistically significant.

Of the eight preventive measures inquired about, “repositioning depending on patient condition” and “pressure injury assessment on admission” were provided to 67.5% and 65.5% of patients, respectively. By contrast, “daily reassessment of risk for all patients” was provided to only 11 patients (5.4%). This makes it the second-least frequently applied preventive measure after “using air mattress” (1.5%). Again, there were statistically significant differences between hospitals regarding all preventive measures provided, except “daily reassessment of risk for all patients”.

**Table 3:** Descriptive statistics of preventive measures provided

	n (%)		*p*
**Number of preventive measures provided(***n* **=** **203,** %**=100)**			< 0.001^†^
1	34	(16.7)	
2	35	(17.2)	
3	49	(24.1)	
4	44	(21.7)	
5	22	(10.8)	
6	19	(9.4)	
Median [IQR]	3	[2–4]	0.019^‡^
**Preventive measures** ^**1**^			
Repositioning depending on patient condition	137	(67.5)	0.029^†^
Pressure injury assessment on admission	133	(65.5)	< 0.001^†^
Pressure-reducing surfaces	129	(63.5)	< 0.001^†^
Manage moisture	86	(42.4)	< 0.001^†^
Optimise nutrition/hydration	79	(38.9)	< 0.001^†^
Daily inspection of skin of at-risk patients	73	(36.0)	< 0.001^†^
Daily reassessment of risk for all patients	11	(5.4)	0.311^†^
Using air mattress	3	(1.5)	< 0.001^†^

n: number; %: percentage; *p*: *p*-value to determine differences between the seven hospitals as regard to the studied variables (statistically significant at *p* ≤.05); IQR: inter-quartile range; 1: Multiple answers are allowed; Statistical test used to determine statistical significance: † Chi-square test; ‡ Kruskal–Wallis test

### Regression analysis

In order to examine the association between patient and PI variables and preventive measures on the one hand, and prevalence rate of PI and HAPI on the other, we performed a correlation analysis. Spearman’s correlation analysis revealed that “pressure injury assessment on admission” has a strong negative correlation (ρ = −0.857) with HAPI prevalence; this was the only variable with a statistically significant correlation (*p* =.014). Hence, we included this variable in a linear regression analysis to predict the magnitude of change in HAPI prevalence resulting from changes in the percentage of “pressure injury assessment on admission”.

In this regression analysis, the changes in the percentage of “pressure injury assessment on admission” account for 70.4% of the variability in HAPI prevalence between hospitals (Table 4). For every 100 patients, a single instance of PI assessment on admission will result in a decrease in the prevalence of HAPI by 0.839%.

**Table 4:** Predictor of HAPI prevalence rate (dependent variable)

	HAPI prevalence rate				
	*R*: 0.839				
	*R*^2^: 0.704				
	B	(SE)	Beta	t	*p*
Constant	12.940	(1.989)		6.507	.001
Pressure injury assessment on admission	−0.091	(0.026)	−0.839	−3.451	.018

*R*^2^: *R*-squared value; B: unstandardised regression coefficient; SE: standard error; Beta: standardised regression coefficient; *p*: *p*-value (statistically significant at *p* ≤.05)

## Discussion

While the results addressed the study objectives satisfactorily, the findings opened our eyes to some aspects that warrant further discussion. Firstly, the mean prevalence of PIs in Kuwait is higher than the global rate [17] and shows more variation. Interestingly, the HAPI prevalence in Kuwait is lower than the global rate but still shows more variation [17]. However, this finding should be interpreted with caution as we are comparing our national rate; which was collected on the medical wards; with the global rate of all wards. What supports this observation is that another recent systematic review and meta-analysis reported a PI prevalence rate of 4.1% (95% CI: 1.3–9.5) on the medical wards [29]. Unfortunately, the study did not indicate whether this rate was for all PIs or the HAPIs only. To conclude, these findings necessitate not only a review of the strategies for preventing and managing PIs nationally, but also to standardise practices across all hospitals. However, further evidence is required before we accept that the significant statistical difference between hospitals is solely due to the variation in their preventive strategies and practices.

The literature has highlighted the role of various intrinsic and extrinsic risk factors in developing PIs [30–34]. Hospitals have many differences in factors that can impact PI prevalence, such as catchment area populations, nationality, age, and number of available beds. Some of these factors might be the cause of what some articles refer to as “unavoidable PIs” [34]. Although determining the extent of unavoidable PIs was beyond the scope of this research, it is useful to keep this concept in mind while addressing the subject of PIs in general. In practical terms, what concerns us in this research is to determine the resources required to overcome non-modifiable factors [32, 35] such as the increase in the number of elderly people or the nationality of patients. Such factors might make re-zoning health regions to control demand, or increasing the number of beds or qualified medical staff to increase capacity, plausible considerations.

Although obesity is one of the principal risk factors for PI [15, 25, 29, 36], it is striking that weight and height—or preferably both, expressed as body mass index (BMI)—were not among the data that is regularly recorded for all patients. A similar national study [37] reported a comparable percentage of patients with PIs who were categorised as underweight (7.1%). On the other hand, the percentages of patients with PIs in normal, overweight and obese groups were significantly different (39.1%, 44.5% and 9.3% respectively) [37]. One cannot make an inference that the differences would stay the same if BMI had been documented in this study for all patients with PIs. Yet, obesity is one of the most important public health concerns In Kuwait [38]. Also, one should not overlook diabetes [39] and hypertension [40], which are both risk factors [15, 29]. If we add these concerns to the ageing population [41], the need for a national public health strategy for controlling the alarming prevalence of obesity and diabetes becomes extremely urgent.

We must not ignore that the prevalence of PIs acquired solely in the community was the largest. In fact, the mean national prevalence of CAPIs is significantly higher than the rate reported by Corbett et al. (7.4%) [22]. This supports our call for developing a national public health strategy. Such a strategy is expected to provide comprehensive home care programmes for the elderly, and to train medical staff and families to prevent, identify and manage PIs.

It is worth considering establishing nursing homes or other long-term care institutions, especially because two-fifths of PIs that occurred in the hospital developed after one month. Whether there was a medical reason (PI or otherwise) for patients to remain in hospital, or because no other appropriate level of care exists, the length of their stays is not commensurate with the acute care that the general public hospitals are supposed to provide. We acknowledge that providing other care institutions might not reduce the number of PIs at national level, but rather transfer some of them from public hospitals to other facilities. Such provision would allow the performance of hospitals in Kuwait to be compared against international standards.

We also noted that more than a third of the PIs were active beyond 3 months. This requires the reasons for the non-response to treatment to be investigated and the current treatment practices to be evaluated. It might be wise to utilise the expertise of wound care specialists, or train nurses on managing PIs that do not respond to treatment.

In stark contrast to the other hospitals, only hospital 4 recorded a majority of PIs related to the use of medical devices (62.1%). This finding warrants particular investigation, especially the contribution of nursing knowledge and attitude, as they are the main care player in PI prevention [42]. It is striking that this hospital had the third-lowest prevalence of PI (13.7%). It is also the only one of the seven hospitals where HAPIs accounted for the larger percentage (89.7%) of the hospital-recorded PIs. However, the last note is not surprising as medical devices are used more in hospitals than at home.

Braden score—another relevant piece of information—was not routinely recorded for all patients in the study. Such practice was reported in the literature. In an observational study conducted on medical wards, Latimer et al. reported that 71.5% of the sample (*n* = 165) in one of the two studied hospitals had not been assessed on admission for risk of PI development [43]. Although hospitals in the current study showed differences between Braden scores and their documentation, the analysis did not indicate any correlation between Braden score and PI prevalence. Based on evidence from two studies, Moore and Patton concluded that they were “*uncertain whether risk assessment using the Braden tool makes any difference to pressure ulcer incidence, compared with training and risk assessment using clinical judgement, or risk assessment using clinical judgement alone*” [44]. Our findings support their conclusion and raise an important question about the value of the overall Braden score; other literature has also questioned its predictive ability and proposed the use of Braden subscales [35, 45, 46].

By contrast, nurses are required to use a form to assess all bedridden patients upon their admission; this is aimed at preventing and evaluating PIs. This was reported in all hospitals (except hospital 4) with a variant compliance rate. Apart from the demographic data and mobility status, the form does not include the items of the Braden tool. Indeed, the form does not use any scoring system. Instead, it contains the following items: continence versus incontinence, the presence of Foley’s catheter, level of consciousness, mental status, and PI (if any) site, size and colour. The main purpose here is to have a baseline assessment of the PI for follow up. Because this preventive measure is the only one that was included in the linear regression analysis for its statistical significance, we can infer that clinically assessing PIs on admission using any tool—regardless of the score—reduces the prevalence of HAPIs.

In contrast to two systematic reviews [17, 29], at our hospitals, stage 2 had the largest percentage of the worst-stage PI groups, not stage 1. This was true of the national distribution and at five of the seven hospitals, regardless of whether the acquisition was in the community or the hospital. This finding raises a significant concern regarding the efficiency and efficacy of the current practices used for early identification and prevention of PIs, especially because almost half of the patients received three or four preventive measures. Notably, the study did not investigate the practices of managing PIs already developed. Unlike for the PI stage, our results align with other studies [12, 14, 17, 29], which reported sacrum, buttocks and heels as the most common locations for PI development.

Many interventions are designed to prevent PIs by reducing friction and shear. These include support surfaces (e.g., mattresses, integrated bed systems, overlays, cushions), repositioning, nutritional supplementation, skin care (e.g., dressings, incontinence management) and topical creams [47]. Although almost half of the patients with PIs were given three to four preventive measures, only three patients received an air mattress. Such a simple provision should not be an issue for a high-income country like Kuwait. In Australia, 24.7% of the study sample (*n* = 799) received an air mattress [48]. The percentage is even higher in the “at PI risk” groups (29.1%). Notably, the study reported PI prevention in routine clinical practice.

Another preventive measure that was expected to be provided to all patients was repositioning (depending on the patient’s condition). Chaboyer et al. [48] found that the repositioning schedule was implemented in 67.4% of the ”at PI risk” groups; the same percentage of this study. This intervention primarily depends on sufficient staffing and appropriate workload assignment. Like the rest of the world, Kuwait suffers from a shortage of nurses [41]. The situation is worsened by the fact that the approved staffing ratios for nurses, devised in 2013 [49], are no longer in line with the responsibilities given to nurses that have increased since. Given the increasing number of bedridden and long-staying patients, staffing ratios should be urgently updated and take workload assignment into account.

### Strengths and limitations

This study has several strengths. It is the first nationwide study in Kuwait or the region to assess PI prevalence in public hospitals. The cross-sectional design enabled different variables in the population sample to be measured at a single point in time for gathering accurate data that are less prone to the potential bias of case series and case reports [50].

However, some limitations exist. The study is designed to determine relationships between variables, not to imply causality between them. In addition, it was not feasible to collect data on the whole sample population (*n* = 1,186). This prevented us from comparing study variables between the PI group and the PI-free group. Furthermore, data were extracted from patients’ files. The validity of such data is subject to the accuracy of the nurses in documenting for the files.

### Practice and research implications

The overall objective of this study was to examine the prevalence of PIs and preventive measures in Kuwait’s general public hospitals. In addition to the previously mentioned strategic initiatives, such as the re-zoning of health regions or providing other levels of care, some changes in practice must be introduced.

There is an immediate need to review and standardise the current measures for preventing and treating PIs. Also, the daily assessment of PIs should include a reassessment of the risk of developing a new PI. In addition, nutritional assessment is currently not undertaken. The patient might require nutritional assessment and support even if they are not underweight. Prolonged confinement to bed results in muscle wasting and a negative nitrogen balance [51], which affects skin integrity and impairs healing processes. We also call for an increase in the number of nurses or providing nursing assistants to carry out duties that do not require nursing competencies, such as repositioning patients.

We call on researchers to study the socio-economic impact of PI on individuals and the healthcare system. There is a gap in the literature regarding PI prevalence and preventive practices in other hospital wards or at tertiary or private hospitals in Kuwait. Another avenue for research is to examine the current practices for treating PIs. Finally, we recommend the use of other research approaches and study designs—such as qualitative approaches and survival analysis—to overcome some of the study limitations and better understand the topic.

## Conclusions

The prevalence of PI in Kuwait is higher than the global rate. This is also the case for the national prevalence of HAPI and CAPI when compared with the global rates. Nationally, the higher percentage of PI acquired purely in the community requires attention. Many risk factors for the development of pressure injuries are public health concerns in Kuwait and effective strategies to address them are needed. The results show many statistically significant differences between hospitals. The patient length of stay is relatively long and is not commensurate with the acute care service. Providing other levels of care might be a necessity.

The PI prevention practices are found to be unacceptable. The majority of patients are not assessed by the Braden tool and the use of air mattresses is almost nil. The management practices of established PI need attention as well. There is a considerable percentage of PIs which persist for months.

Our evaluation of these results is expected to help healthcare leaders in Kuwait to better visualise the problem and set realistic targets for improvement. As such, the findings of this study should enlighten and lead national strategies aimed at reducing both CAPI and HAPI. This topic is still relatively young in Kuwait and researchers are encouraged to explore all its aspects, using a variety of research methodologies and study designs.

### Electronic supplementary material

Below is the link to the electronic supplementary material.

Supplementary Material 1

## Data Availability

The datasets generated and/or analysed during the current study are not publicly available owing to MOH restrictions. However, datasets are available from the corresponding author upon reasonable request.

## Abbreviations

B: Unstandardised regression coefficient
Beta: Standardised regression coefficient
BMI: Body mass index
CAPI: Community-acquired pressure injury
CI: 95% Confidence interval
EPUAP: European Pressure Advisory Panel
HAPI: Hospital-acquired pressure injury
IQR: Inter-quartile range
n: Number
NPUAP: National Pressure Ulcer Advisory Panel
PI: Pressure injury
PPPIA: Pan Pacific Pressure Injury Alliance
SD: Standard deviation
SE: Standard error
STROBE: Strengthening the Reporting of Observational studies in Epidemiology
