# Supplementary material for: Prevalence of pressure injury on the medical wards of public general hospitals in Kuwait: a national cross-sectional study
Source: BMC Health Serv Res. 2024 Feb 7;24:173. doi: 10.1186/s12913-024-10615-x (PMC10851550; doi:10.1186/s12913-024-10615-x)
Supplement: Supplementary file 1 — Supplementary Material 1 [file 12913_2024_10615_MOESM1_ESM.pdf]

Data Collection Form for Prevalence Rate of Pressure Injury

Please fill in this form for every patient with pressure injury

dr.hossam.elamir@gmail.com Switch account

Not shared

* Indicates required question

Hospital name *

Your answer

Ward number *

Your answer

Patient's file number *

Your answer

Sex *

- Male
- Female

Patient age in years *

Your answer

Patient weight in kg *

Your answer

Patient height in cm *

Your answer

Date of current admission *

Date

mm/dd/yyyy

Mobility condition/Activity *

- ○ Mobile
- ○ Immobile
- ○ Mobile with assistant

Patient has *

Select all that apply

- ☐ Chronic disease
- ☐ Spinal cord injury
- ☐ Immobility/Reduced mobility
- ☐ Vasopressor infusion
- ☐ Sepsis
- ☐ Dehydration
- ☐ Other:

Date of pressure injury onset *

Date

mm/dd/yyyy

Pressure injury is *

- ◯ Hospital-acquired
- ◯ Community-acquired
- ◯ Hospital and community-acquired

Location/s of pressure injury *

Select all that apply

- ☐ Sacrum
- ☐ Buttocks
- ☐ Heels
- ☐ Back
- ☐ Back of head
- ☐ Elbows
- ☐ Other:

Number of pressure injuries *

Your answer

Worst-stage pressure injury *

- ◯ Stage 1
- ◯ Stage 2
- ◯ Stage 3
- ◯ Stage 4
- ◯ Suspected deep tissue injury

Is pressure injury related to medical devices? *

- ◯ Yes
- ◯ No

Preventive Measurement Taken *

Select all that apply

- ☐ Pressure injury assessment on admission
- ☐ Reassess risk for all patients daily
- ☐ Inspect skin of at-risk patients daily
- ☐ Manage moisture
- ☐ Optimise nutrition/hydration
- ☐ Pressure-reducing surfaces
- ☐ Repositioning depends on their condition
- ☐ Using air mattress
- ☐ Other:

Braden scale assessment score documented? *

- ◯ Yes
- ◯ No

Braden scale assessment score *

Your answer

Submit

Clear form

Never submit passwords through Google Forms.

This content is neither created nor endorsed by Google. Report Abuse - Terms of Service - Privacy Policy

Google Forms
